# Gene regulation helps species thrive in new climates

**DOI:** 10.1038/s42003-023-05755-5

**Published:** 2024-01-11

**Authors:** Christina Paliou

**Affiliations:** Centro Andaluz de Biología del Desarrollo (CABD), Consejo Superior de Investigaciones Científicas (CSIC)/ Universidad Pablo de Olavide (UPO)/ Junta de Andalucía, Seville, Spain

## Abstract

Despite ample investigation on *cis*-regulatory alterations in evolution, the contribution of these effects to environmental adaptation is poorly understood. Ballinger and colleagues dissected the *cis*- and *trans*-regulatory impact on gene expression associated with the adaptation of house mice to temperate and tropical climates, highlighting potential mechanisms acting at a short evolutionary timescale.

The key players controlling gene expression in organisms are *cis*-regulatory elements, such as genetic enhancers, and *trans*-activating elements, like transcription factors, that then bind *cis*-regulatory elements. Gene regulatory networks depend on the combination of genes, *cis*- and *trans*-regulatory elements, and changes in any of these individual factors can lead to transcriptomic alterations that rewire these networks. Eventually, these transcriptomic changes can result in the emergence of new traits across evolutionary timescales^1^. While transcriptomic divergence is often attributed to genetic changes, environmental influences can also drive changes in gene expression. Understanding the mechanisms behind organismsʼ responses to environmental changes will enrich our knowledge on how populations adapt to new habitats.

A relevant animal model for investigating short-term adaptation are house mice, since they moved from Western Europe to America roughly 500 years ago^2^. While previous studies focused on genetic differences affecting the mouse morphology, physiology, and behavior across different climates, the influence of the environment on these traits was not considered per se. In a recent study, Ballinger and colleagues set out to elucidate how gene regulation has shaped the rapid adaptation of two natural populations of house mice (*Mus musculus domesticus*) in extremely different thermal environments, New York (USA) and Amazonas (Brasil)^2^.

First, the authors assessed the transcriptome of tissues known for their role in body temperature regulation (brown adipose tissue and liver) in male and female mice originating from Brasil and New York, housed in warm (21 °C) and cold (5 °C) environments (Fig. 1a). This setup enabled the authors to assess the influence of genotype, represented by geographic origin, as well as the contribution of temperature conditions on gene expression. The transcriptomic comparison in both tissues shows that mice largely cluster by their location of origin and only 5–10% of genes were differentially expressed in response to temperature. Next, comparing the gene expression differences between Brasil and New York mice to those observed in Brasil mice raised in warm vs. cold environments, showed high correlation, particularly in the liver. This finding suggests that the environmentally driven changes in gene expression could then shape adaptation in those conditions. Second, the authors performed allele-specific expression experiments, to investigate what percentage of the expression divergence between New York and Brasil mice were due to *cis*- *vs. trans-*effects, and whether these changes are influenced by the environment. Specifically, any changes in gene expression between alleles of the hybrid mice were considered as a *cis*-effect, whereas *trans*-effects were defined as differences in gene expression that were only observed between parental mouse lines (and not hybrid offspring) (Fig. 1b, c). The authors found that *cis*-regulation contributes more to the expression divergence between New York and Brasil mice and is largely resistant to any influence of temperature. On the contrary, thermal conditions affect more the *trans*-regulation, meaning that *trans*-effects might underlie the ability of genes to respond rapidly to environmental changes. Finally, the authors found significant overlap between genomic sequence variation with *cis*-regulated genes among wild mice populations living in cold climates, but not in warm climates. This finding hints that natural selection preferably acted on *cis*-regulated genes related to metabolism or body weight, which might explain the rapid adaptation of house mice to cold environments.

**Fig. 1:**
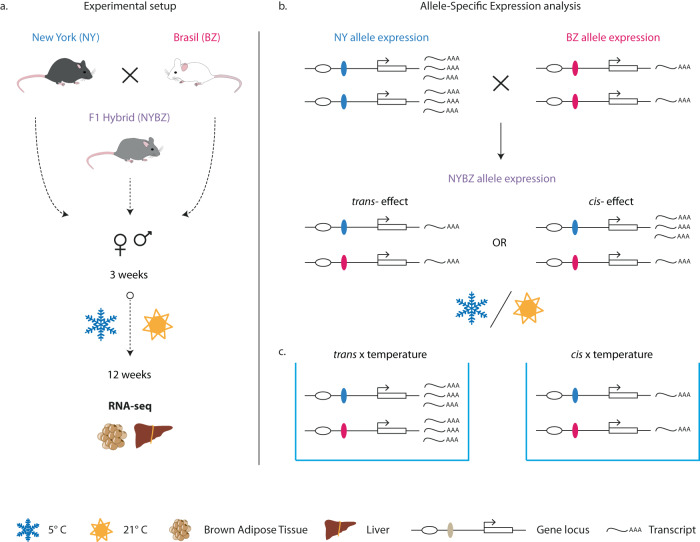
Schematic representation of the experimental setup in Ballinger et al.^2^. **a** New York (NY) and Brasil (BZ) mice are housed in 21 °C and bred to generate F1 hybrids (NYBZ). 3-week-old full siblings and F1 hybrids are split into groups that are kept under cold (5 °C) or warm (21 °C) treatments until the 12th week of age. Then, brown adipose tissue and liver are processed for transcriptomic analysis by RNA-seq. **b** Allele-specific expression analysis disentangles whether parental transcriptomic differences are due to *cis-* or *trans*-effects. Differential allelic expression of a gene in the F1 hybrids indicates one or many *cis*-regulatory alterations, inherited by each of the parents in the same *trans*-environment. A *trans*-effect is speculated when no allele-specific expression is detected in hybrids, but differences are observed in the parental gene expression. **c** Exposing parental lines and the F1 hybrids to different temperatures is a means to additionally assess the environmental influence on gene expression. This figure was adapted from Ballinger et al.^2^.

Overall, the work from Ballinger et al. highlights the role of *cis*-regulation in adaptive evolution, while suggesting *trans*-factors might be the first step in enabling species to colonize new habitats. Nevertheless, a limitation of this study is that the complicated relationship of plasticity, selection and adaptation is only briefly discussed. Future research could dive more into the contribution of individual *cis*- or *trans*-elements, how they fit into specific gene regulatory networks, and the overall molecular mechanisms through which they could orchestrate divergence in gene expression. In combination with fitness experiments that would assess how these changes affect the ability of mice to survive and reproduce, it could eventually be predicted whether these elements can play a role in adaptive evolution. Given the increasing relevance of climate change, a better understanding of the key players and the processes underlying adaptation to new environments is critical and can be achieved by bridging the gap between molecular and evolutionary biological studies.
